# Evaluation of the performance of advantage P.f. malaria Card^®^ and advantage malaria Pan + Pf Card^®^, two rapid diagnostic tests for parasitological confirmation of malaria cases in field situation in Togo

**DOI:** 10.1186/s13071-023-06062-y

**Published:** 2023-11-30

**Authors:** Diwaba Carmel Teou, Ameyo Monique Dorkenoo, Essoham Ataba, Smaila Alidou, Kossi Yakpa, Agueregna Abdou-Kerim, Issaka Maman, Amegnona Agbonon

**Affiliations:** 1 Faculté Des Sciences, Université de Lomé, Boulevard Eyadema, 01BP 1515 Lomé, Togo; 2 Faculté Des Sciences de La Santé, Université de Lomé, Boulevard Eyadema, 01BP 1515 Lomé, Togo; 3 Ministère de la Santé de L’Hygiène Publique et de L’Accès Universel Aux Soins, Programme National de Lutte Contre Le Paludisme, Quartier Administratif, 01BP 518 Lomé, Togo; 4 Département de Santé Publique, Unité de Formation et de Recherche en Sciences de la Santé, Université Joseph KI-ZERBO, Ouagadougou, Burkina Faso; 5 Ministère de la Santé de L’Hygiène Publique et de L’Accès Universel Aux Soins, Institut National d’Hygiène, Quartier Administratif, 01BP 1396 Lomé, Togo

**Keywords:** RDT, Malaria, Performance, Microscopy, PCR

## Abstract

**Background:**

In Togo, malaria remains a major public health problem, and the management of suspected cases requires confirmation with appropriate biological methods. Malaria diagnosis has been improved by the introduction of rapid diagnostic tests (RDTs), recommended by the World Health Organization (WHO) for areas where microscopy is not available. To be used, these RDTs must meet performance criteria defined by the WHO. This study was conducted to evaluate the diagnostic performance of two RDTs: Advantage P.f. Malaria Card^®^ detecting HRP2 antigen and Advantage Malaria Pan + Pf Card^®^ detecting both HRP2 and pLDH antigens.

**Methods:**

This was a cross-sectional analytical study conducted from December 2019 to February 2020 on malaria-suspected cases received in three sentinel sites in Togo and from whom capillary blood was collected to perform the two RDTs according to the manufacturer's instructions. Sensitivity and specificity were estimated by comparing to thick/thin blood smear, the gold standard, and to PCR, which is a more sensitive.

**Results:**

A total of 390 participants (54.9% female) with a median age of 18 (± 0.8) years were included in the study. The sensitivity of both Advantage P.f. Malaria Card® and Advantage Malaria Pan + Pf Card^®^ compared to thick/thin blood smear was 91.8% and 91.3%, respectively, and for both the specificity was 94.7%. Compared to PCR, the sensitivity was 84.2% and 83.8%, respectively, and the specificity 96.5%.

**Conclusions:**

The performances of the Advantage P.f. Malaria Card^®^ and Advantage Malaria PAN + Pf Card^®^ compared to microscopy, considered the gold standard, were acceptable under the field conditions found in Togo. They can therefore be used for the biological diagnosis of malaria.

**Graphical Abstract:**

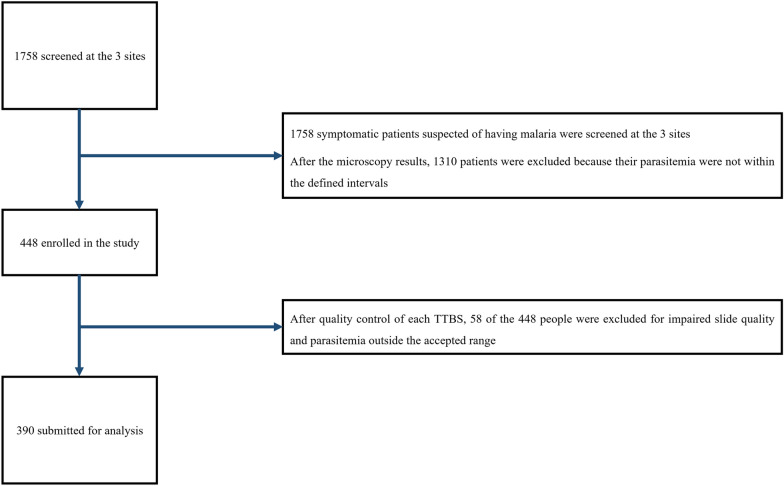

## Background

Malaria, one of the major endemic tropical diseases, is a febrile erythrocytopathy caused by a parasite of the genus *Plasmodium* and transmitted to humans by infected female *Anopheles* mosquitoes. In 2021, the World Health Organization (WHO) estimated the worldwide number of malaria cases to be 247 million with 619000 deaths attributable to malaria, 95% of which occurred in the African region [1].

In Togo, malaria remains a public health problem, with children and pregnant women being the most affected. Indeed, its prevalence over the period from 2017 to 2019 in children aged 6 to 59 months was 28%, and the percentage of individuals infected with malaria who died decreased from 3.2% in 2019 to 2.1% in 2021 [2].

Among the control strategies adopted to reduce morbidity and mortality linked to this parasitic disease, the malaria treatment policy has emphasized the early (within 24 h) and effective management of confirmed cases. In accordance with WHO recommendations, uncomplicated case management should be based on confirmatory biological diagnosis and prompt treatment using artemisinin-based combination therapy (ACT) [3].

The gold standard method for biological confirmation of malaria remains the thick/thin blood smear (TTBS), which demonstrates the different stages of the plasmodial species. However, the reliability of its results is highly dependent on the technical platform, the skills of the microscopist, and the reading time and requires the implementation of a quality management system [4].

For more than a decade of the development of rapid diagnostic tests (RDTs) with the support of partners, malaria diagnosis has improved considerably, as biological confirmation can be done using these RDTs. The main advantages are the ease of use and the fact that they do not require electricity or equipment. In addition, results can be obtained in 15–30 min [3]. Based on the immunochromatography technique, they detect *Plasmodium*-specific antigens by immunocapture using antibodies, which can be specific to a single species or "pan-specific," capable of recognizing multiple plasmodial species. Histidine-rich protein 2 (HRP2), plasmodium lactate dehydrogenase (pLDH), and less frequently aldolase are the proteins mainly used for commercially available RDTs. To ensure the quality of these RDTs for use in countries, in 2006, the WHO and the Foundation for New and Innovative Diagnostic Tools (FIND) initiated a process of systematic evaluation and comparison of the effectiveness of commercially available malaria RDTs [5].

Furthermore, numerous evaluations of these RDTs have demonstrated high levels of performance compared with TTBS as the gold standard [6–8]. Thus, the objective of this study was to compare the performance of the Advantage P.f. Malaria Card^®^ and Advantage Malaria PAN+Pf Card^®^ compared to microscopy, considered the gold standard, and to PCR, which is more sensitive.

## Methods

### Study design and sites

This was a cross-sectional analytical study that took place from December 2019 to February 2020 in Togo. Malaria transmission is stable throughout the country, with two predominant climates: the sub-equatorial with two rainy seasons in the southern part of the country and the tropical with a single rainy season in the northern part. Thus, three sentinel sites for monitoring the effectiveness of ACTs used for the treatment of uncomplicated malaria were used for this evaluation. The Social Medical Center (SMC) of Cacaveli, a public health facility in Lomé, the capital of Togo, was the first site, to which the SMC "UTB Circulaire" was added because of the relatively low patient attendance at the site. The SMC Ahépé, a public health facility in the Maritime region, was the second site, located 66 km from Lomé, to which the hospital "la Providence de Kouvé" was also added. The last site was the Sokodé polyclinic located in the central region approximatively 340 km north of Lomé. The first two sites were sub-equatorial, located respectively in urban and rural areas, and the third site was tropical, located in a semi-urban area.

### Study population and sampling

The study population was symptomatic patients suspected of having malaria who were seen in consultation at the different sentinel sites and for whom TTBS was prescribed. Since the sensitivity of RDTs varies according to the parasite density [9, 10] and can reach 100 parasites/μl, which may vary from one product to another [11], blood smear-positive subjects were divided into two groups, a low parasite density group (patients with asexual parasitemia count per microliter between 50 and 1000) and a high parasite density group (those with asexual parasitemia per microliter between 2000 and 10,000). The control group comprised subjects negative for any species of *Plasmodium*.

### Sample size

The sample size calculation methods of Buderer et al*.* [12] were used. For calculation of sensibility and specificity, we used the formula Np = $\frac{{{Z}_{a/2}}^{2}\mathrm{se}(1-se)}{{E}^{2}}$ to estimate the number of cases (positives) to include and the formula Nn = $\frac{{{Z}_{a/2}}^{2}\mathrm{sp}*(1-sp)}{{E}^{2}}$ to estimate the number of controls (negatives). A 90% sensitivity was estimated for low parasitemia and 95% for high parasitemia with a tolerated margin of error (E) of 5% and an accepted risk of error (α) of 5% (Zα/2 at 1.96); the size of positives with low parasitemia for inclusion was 139, and the number of positives with high parasitemia for inclusion was 73. For specificity estimated to 90%, the number of included controls was 139. Therefore, this study should include a total sample size of 351 participants.

### Inclusion and non-inclusion criteria

Inclusion criteria were considered by group. Included in the low parasitemia group were patients with asexual parasitemia count per microliter between 50 and 1000; in the high parasitemia group, those with asexual parasitemia per microliter between 2000 and 10000; and in the control group, individuals with negative thick blood smear [13–15]. Signed written consent was obtained from each adult patient and the parent/guardian of the children before their enrollment in the study. Any person who did not meet the above criteria and who voluntarily declined to participate in the study was not included in the study population.

### Data collection

A structured questionnaire was used to collect information on sociodemographic characteristics, clinical signs presented, history of the disease, and existence of other diseases if applicable.

### Laboratory tests

Each patient had a capillary blood sampling for a TTBS. After the microscopy results were known (having parasitemia within a certain range or being malaria negative), a second sample was taken from the included subjects to test the RDTs evaluated for *Plasmodium* spp. infection, and dried blood spots (DBS) were performed on Wattman type III paper.

### Thick and thin blood smear

The thick and thin smears were made on the same slide. Two slides were made. After fixing the thin blood smear with methanol for a few seconds, the first slide was stained by Giemsa 10%, 10 to 15 min, for initial screening (having parasitemia within a certain range or being malaria negative), and the second at 3%, 45 min for detailed examination to obtain definitive results. [16]. After drying, the slides were then read under an immersion microscope with an 100× objective to determine the positivity and identify the plasmodial species and estimate the parasitemia.

### Rapid diagnostic tests

Two types of Advantage brand RDTs were evaluated: Advantage P.f. Malaria Card^®^ (IR016025), which is specific for *Plasmodium falciparum*, and Advantage Malaria Pan + Pf Card^®^ (IR231025), which can detect *P. falciparum*, *P. malariae, P. vivax*, and *P. ovale* (J. Mitra & Co. Pvt. Ltd.). Both RDTs are based on the immunochromatographic technique: Advantage P.f. Malaria Card®, using a monoclonal anti-Pf HRP2 antibody, detects HRP2 antigen, specific for *P. falciparum*, and Advantage Malaria Pan + Pf Card^®^, in addition to the *P. falciparum*-specific anti-Pf HRP2 monoclonal antibody, detects pLDH (plasmodium lactate dehydrogenase) antigen, which is common to all plasmodial species, using a *Plasmodium*-specific anti-Pan pLDH monoclonal antibody. Both RDTs were performed simultaneously in the laboratory by study staff for each enrolled subject according to the manufacturer's instructions as well as the interpretation of the results. Four µicroliters of fresh blood from finger prick using the inverted up (by touching the base of the inverted cup into the blood drop) was immediately placed in the sample well, and then three drops of the assay buffer were added to the buffer well. The results were read at 20 min.

### Real-time PCR assay

*Plasmodium* DNA was extracted by the heating method described by Miura et al. [17]; the Qiagen^®^ kit was used according to the manufacturer's instructions to validate the heating extraction method. Real-time PCR was performed using primers, probes, and reaction conditions described by Shokoples et al. [18] and Divis et al. [19] with the following modification; the fluorophores for the *P. falciparum* probes were replaced with Cy5-BHQ-1 [20]. Two separate reactions were performed: (i) a screening reaction for the presence of *Plasmodium* species with *Plasmodium* genus-conserved primer pair (Plasmo1 and Plasmo2) and Plasprobe to detect a conserved region of the *Plasmodium* 18S ssu rRNA gene of all five human malaria parasites [21]; (ii) a monoplex PCR for the detection of *P. falciparum* using species-specific forward primer paired with Plasmo2 and species-specific probes [18]. Briefly, the screening and monoplex assays were performed with a final volume of 25 μl containing 5 μl template DNA, 12.5 μl QuantiFast Multiplex PCR master mix (Qiagen, Germany), and 7.5 μl pooled primer and probe mix. All assays were performed under standard conditions (1 cycle of 95°C for 5 min; 45 repeated cycles of 95°C for 30 s and 60°C for 30 s) with the CFX96 Real-time PCR machine (Bio-Rad, USA).

### Quality control

Duplicated reading was done by two experienced microscopists for each TTBS [22]. If the coefficient of variation in parasite density estimate was > 5%, a third reading was performed by another independent microscopist [16]. If there was a difference between the study sites' parasitemia and results found by the quality control, parasitemia of the quality control was considered. The estimated parasitemia was used to constitute the three groups.

RDT results are considered valid and interpreted only in the presence of the control line at the end of the test, following the manufacturer's instructions.

A cutoff of 40 cycles was used to define PCR-positive samples. The test panel included several controls: (i) negative sample extraction as a negative control, (ii) β2-macroglobulin (β2M) target amplification (cycle threshold < 40) as a positive extraction control for the sample, and (iii) a positive reference control to detect any variation between runs and non-template control for each of the master mixes [20, 21].

Training of site team members and supervisors was conducted to standardize work methods and ensure the smooth running of the activity, especially for filling out questionnaires and conducting RDTs, TTBS, and filter paper sampling.

### Endpoints

Thick/thin blood smear and PCR were considered the reference methods in this evaluation to which both RDTs were compared. Sensitivity, specificity, positive predictive value (PPV), and negative predictive value (NPV) are the estimated performance indicators of these two RDTs. Sensitivity and PPV were calculated for low and high parasitemia.

### Data management and analysis

Data were recorded on register forms, entered in a Microsoft Excel database (Microsoft Corp, Redmond, Washington, USA), and analyzed using EpiInfoTM version 3.5.1 software. Sensitivity, specificity, and positive and negative predictive values of RDTs were determined using microscopy (or PCR), using 2 × 2 contingency tables. Exact 95% confidence intervals (95% CI) were calculated for each measure listed above.

### Ethical considerations

The study protocol obtained ethical clearance from the Bioethics Committee for Health Research (CBRS) of Togo (no. 046/2019/CBRS of November 21, 2019) before its implementation. In addition, signed consent was obtained from adults and children’s parents/guardians. Any patient detected positive by at least one of the methods was referred to the clinicians at the sites for free management with an antimalarial drug available through the National Malaria Control Program.

## Results

### Characteristics of study participants

**Fig. 1:**
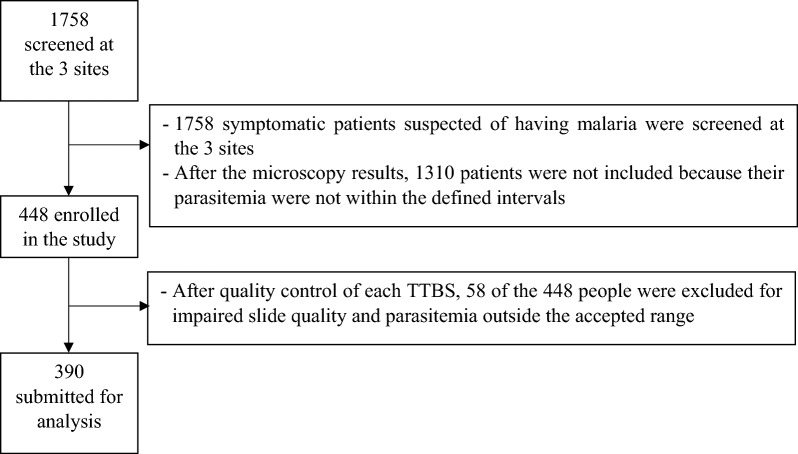
Flow diagram for selection

Of the 1758 people screened at the three sites, 448 were enrolled in the study, for an inclusion rate of 25.5%. Fifty-eight were excluded for quality control (impaired slide quality and parasitemia outside the accepted range). The final number submitted for analysis was 390 (Fig. 1).

The mean age of those included was 18 (± 0.84) years with extremes of 10 months and 85 years and a sex ratio (M/F) of 0.8. The main reason for consultation was fever (61.0%). The sociodemographic characteristics of the participants are summarized in Table 1. The evaluation of the Advantage P.f. Malaria Card^®^ and Advantage Malaria Pan + Pf Card^®^ compar with microscopy, considered 390 patients, of which 164 were in the control group, 142 in the low parasitemia group, and 84 in the high parasitemia group (Table 2). Details of the numbers used to compare the two RDTs against microscopy and PCR are provided in Table 3.

**Table 1:** Patients' sociodemographic characteristics and clinical signs

	Frequency (*n*)	Percentage (%)
Age (years)		
0–5	111	28.5
6–9	36	9.2
10–14	55	14.1
15–19	55	14.1
≥ 20	133	34.1
Gender		
Female	214	54.9
Male	176	45.1
Clinical symptoms		
Fever	238	61.0
Headache	147	37.7
Asthenia	85	21.8
Anorexia	68	17.4
Vomiting	52	17.4
Nausea	32	8.2
Chills	29	7.4
Coughing	20	5.1
Abdominal pain	19	4.9
Diarrhea	6	1.5

**Table 2:** Total number of samples included by defined sites

Sites	Lomé	Kouvé	Sokodé
	*n* (%)	*n* (%)	*n* (%)
Microscopy (TTBS)			
Negative: *N* = 164	54 (13.8)	51 (13.1)	59 (15.1)
Positive			
Low parasitemia: *N* = *142*	27 (06.9)	97 (24.9)	18 (04.6)
High parasitemia: *N* = *84*	27 (06.9)	38 (09.7)	19 (04.9)
Total (*n* = 390)	108 (27.7)	186 (47.7)	96 (24.6)
PCR			
Negative (*n* = 143)	63 (16.4)	24 (6.3)	56 (14.6)
Positive (*n* = 240)	58 (15.1)	139 (36.3)	43 (11.2)
Total (*n* = 383)	121 (31.6)	163 (42.6)	99 (25.8)

**Table 3:** RDTs' diagnostic classification using TTBS and PCR as the reference methods

	Microscopy as reference method						PCR as reference method	
	Low parasitemia^a^		High parasitemia^a^		Total			
	Positive	Negative	Positive	Negative	Positive	Negative	Positive	Negative
Advantage Malaria P.f. card®								
Positive	125	8	76	9	201	9	202	5
Negative	16	157	2	161	18	162	38	138
Advantage Malaria Pan + Pf Card								
Positive	124	8	76	9	200	9	201	5
Negative	17	157	2	161	19	162	39	138

^a^Low parasitemia = 50–100 asexual parasitemia/µl, high parasitemia = 2000–10000 asexual parasitemia/µl

### Performance of RDTs evaluated compared to microscopy

Of the 226 microscopy-positive cases, 96.9% (219/226) were *P. falciparum*, 2.2% (5/226) *P. malariae*, and 0,9% (2/226) *P. ovale*. The sensitivity and specificity of the Advantage P.f. Malaria Card® compared to TTBS for the detection of *P. falciparum* were 91.8% [CI = 87.1–94.9] and 94.7% [CI = 90.0–97.4], respectively; PPV and NPV values were 95.7 [CI = 92.2–97.7] and 90.0 [CI = 85.2–93.3] (Table 4). The sensitivity and specificity of the Advantage Malaria Pan + Pf Card® RDT were 91.3% [CI = 86.6–94.6] and 94.7% [CI = 90.0–97.4], respectively; PPV and NPV values were 95.7 [CI = 92.2–97.7] and 89.5 [CI = 84.7–92.9] (Table 4).

**Table 4:** Performance levels of RDTs evaluated against TTBS and PCR for the detection of *Plasmodium falciparum*

	Advantage Malaria P.f. card®	Advantage Malaria Pan + Pf Card®
Microscopy as reference method		
Low parasitemia^a^		
SE^b^ [95% CI]	88.7 [81.9–93.2]	87.9 [81.1–92.7]
PPV^b^ [95% CI]	94.0 [88.8–96.9]	93.9 [88.7–96.8]
High parasitemia^a^		
SE^b^ [95% CI]	97.4 [90.2–99.6]	97.4 [90.2–99.6]
PPV [95% CI]	89.4 [81.7–94.1]	89.4 [81.7–94.1]
*Total*		
SE [95% CI]	91.8 [87.1–94.9]	91.3 [86.6–94.6]
SP^b^ [95% CI]	94.7 [90.0–97.4]	94.7 [90.0–97.4]
PPV [95% CI]	95.7 [92.2–97.7]	95.7 [92.2–97.7]
NPV^b^ [95% CI]	90.0 [85.2–93.3]	89.5 [84.7–92.9]
PCR as reference method		
SE [95% CI]	84.2 [84.7–92.8]	83.8 [78.5–88.2]
SP [95% CI]	96.5 [92.0–98.9]	96.5 [92.0–98.9]
PPV [95% CI]	97.6 [94.4–99.0]	97.6 [94.4–99.0]
NPV [95% CI]	78.4 [71.5–84.1]	78.0 [71.0–83.6]

^a^Low parasitemia = 50–100 asexual parasitemia/µl, high parasitemia = 2000–10000 asexual parasitemia/µl

^b^*SE* = sensitivity, *SP* = specificity; *PPV* = positive predictive value; *NPV* = negative predictive value

### Performance of RDTs evaluated compared to PCR

For the PCR analysis, detection of plasmodial DNA of *P. falciparum* was inconclusive for seven samples; therefore, the estimation of the performance of the two RDTs evaluated compared to PCR was done on a total of 383 patients divided into 143 for the PCR-negative group and 240 for the *P. falciparum* PCR-positive group (Table 2). The sensitivity of the Advantage P.F Malaria Card^®^ RDT compared to PCR for *P. falciparum* infection detection was 84.2% [CI = 78.9–88.6], and the specificity was 96.5% [CI = 92.0–98.9]. This sensitivity was 83.8% [CI = 78.5–88.2] for the Advantage Malaria PAN + P.F Card^®^ RDT while specificity was the same (96.5%). The PPV and NPV values are shown in Table 4.

## Discussion

The World Health Organization recommends that malaria-endemic countries and their partners procure pre-qualified RDTs for the biological confirmation of suspected malaria cases. As such, the FIND group conducts regular evaluations and makes the results available to countries. The Advantage P.f. Malaria Card^®^ and Advantage Malaria Pan + Pf Card^®^ are WHO pre-qualified RDTs whose performance has been evaluated against clinical wild-type samples containing *P. falciparum* and *P. vivax* at low (200) and high (2000) parasite densities (plasmodia/μl) as well as negative samples for any pathology [15]. J. Mitra and Co., Pvt., Ltd., in the context of the marketing of two new RDTs, wanted these RDTs to be tested in a field-use situation in Togo. This study was therefore conducted at sentinel sites in Togo, where the evaluation of the effectiveness of ACTs is often carried out, thus ensuring that evaluation procedures are respected.

The performance levels of the Advantage P.f. Malaria Card® and Advantage Malaria Pan+Pf Card® for the detection of *P. falciparum* compared to microscopy were 91.8% and 91.3% for sensitivity, respectively, and 94.7% for specificity. Our results are similar to other evaluations done that also considered microscopy as a gold standard method. For example, a study in northwest Ethiopia evaluating CareStart™ showed a sensitivity of 92.9 and specificity of 95.4 [23]; another in Central African Republic found a sensitivity of 92.3 for the SD Bioline AgPf^®^(HRP2) RDT [24]. A study by Margiano [25] noted a specificity of 93.56% for the Alere HS RDT^®^.

In our study, density-dependent sensitivity was observed with microscopy as the reference: indeed, it was higher for high parasitemia for both evaluated RDTs (97.4% vs. 88.7% and 87.9%, respectively). This trend corroborates the finding of Wanja et al. [10] who, in an evaluation of the diagnostic performance of four types of RDTs targeting HRP2 (2016) in Kenya, noted that the overall sensitivity, which was > 90% for the four RDTs evaluated, fell below 90% for parasitemia < 200 P/µl (sensitivity between 79 and 89%). These data confirm the results of the study conducted in Togo by Dorkenoo et al. [9] as part of the evaluation of the performance of actors in malaria diagnosis by RDTs, where dried tube samples of *P. falciparum* of 100 to 200 parasites/µl were not reliably detected by the RDTs used.

This limitation of RDT detection for low parasitemia [26] would explain the false-negative cases observed in our study, although other factors have been listed by other authors including the lack of specificity of the monoclonal antibodies used to design RDTs, the genetic diversity of the HRP2 parasite antigen, or the deletion of the gene-coding HRP2 [27]. Recently, studies have reported false-negative RDTs primarily because of the deletion of the histidine-rich protein (*fhrp2* and *pfhrp3*) genes in field isolates of *P. falciparum* [28].

In our study, the RDTs evaluated showed a relatively high specificity (94.7%) compared to microscopy. Indeed, WHO recommends that RDTs used for biological confirmation of malaria should have at least a specificity > 90% [29]. The low false-positive rate (5.3%) found in our study could be related to the possible presence of rheumatoid factors in these patients [30] and/or to the persistence of HRP2 in the blood several days after parasite clearance. Indeed, Grandesso et al. [31] showed in their study that the median time to become negative was ≥ 35–42 days for the HRP2 tests. Another study supporting this finding has shown that this persistence can be up to 52 days [32].

Significant positive and negative predictive values were found for these RDTs compared to TTBS in this study. Thus, the high PPV for both RDTs (95.7%) reinforces the sensitivity and specificity data found for these RDTs to be used for biological confirmation of malaria cases in malaria-endemic areas. The NPVs of the Advantage P.f. Malaria Card^®^ and Advantage Malaria Pan+Pf Card®, 90.0 and 89.5%, respectively, are probably related to the time of our study, which started at the end of the rainy season, while the evaluations of the other authors were done during the period of high transmission. Indeed, the predictive values (PPV and NPV) are a function of the prevalence of the disease in the study area [33].

Although microscopy is the gold standard detection technique for malaria, the performance of these RDTs was also estimated with PCR, a technique with better sensitivity than TTBS. The performance of the RDTs was slightly lower than that obtained when considering TTBS. The sensitivity of the Advantage P.f. Malaria Card® and Advantage Malaria Pan+Pf Card^®^, which were 91.8% and 91.3%, respectively, compared to microscopy, fell below 90% when PCR was used as a reference method (84.2% and 83.8, respectively). Indeed, PCR can detect parasites at levels as low as 0.002 P/µl, implying that the lower sensitivity range observed could be due to sub-microscopic infections in the population. Also, Matangila et al. [34] showed that 65% of the microscopy-negative samples analyzed by PCR were submicroscopic infections.

The opposite was observed for specificity whereby the levels were higher compared to PCR (96.5%) than microscopy (94.7%). This indicates that compared with PCR, they have a higher ability to actually detect a malaria-negative patient. False-positive results could be explained by the persistence of HRP2 circulation in the blood for > 2 weeks even after antimalarial treatment [34]. The different factors such as false positives, false negatives, inability to detect submicroscopic infections, persistent HRP2 antigenemia, and HRP2 polymorphism, considered biases in the interpretation of the real level of RDT performance, would require PCR to be added for an improvement of the quality of its diagnosis [29, 35].

Our study has some limitations. Indeed, although the Advantage Malaria Pan+Pf Card® RDT detects species other than *P. falciparum*, the performance of this RDT to detect other plasmodial species has not been assessed because of the low number of positive cases for these other species. Among the included subjects whose malaria test result was positive according to the results of the microscopic examination, 2.2% had a positive result for *P. malariae* and 0.9% for *P. ovale*.

## Conclusion

The diagnostic performance of the Advantage P.F Malaria Card^®^ and Advantage Malaria Pan + PF Card^®^ RDTs for the parasitological confirmation of malaria cases compared to the microscopy as the gold standard, although varying according to the parasite density, was acceptable under the field conditions found in Togo; even if considering PCR as the reference method, this performance has slightly declined. As sub-microscopic infections are expected to be more frequent, an impact of the control strategies undertaken by Togo, the determination of the performance of the new RDTs should perhaps consider PCR in the evaluation procedure as part of the continuous improvement of these diagnostic tools to achieve the elimination of malaria in the countries where it remains endemic.

## Data Availability

All data used to draw conclusion of the study are provided in the manuscript.
